# Higher oxidative stress in skeletal muscle of McArdle disease patients

**DOI:** 10.1016/j.ymgmr.2017.05.009

**Published:** 2017-06-09

**Authors:** Jan J. Kaczor, Holly A. Robertshaw, Mark A. Tarnopolsky

**Affiliations:** a Department of Pediatrics, McMaster University, Hamilton, Ontario L8N 3Z5, Canada; b Department of Medicine, McMaster University, Hamilton, Ontario L8N 3Z5, Canada; c Department of Neurobiology of Muscle, Gdansk University of Physical Education and Sport, Gdansk, Poland

**Keywords:** McArdle disease, Oxidative stress, Enzyme activity, Skeletal muscle

## Abstract

McArdle disease (MCD) is an autosomal recessive condition resulting from skeletal muscle glycogen phosphorylase deficiency. The resultant block in glycogenolysis leads to an increased flux through the xanthine oxidase pathway (myogenic hyperuricemia) and could lead to an increase in oxidative stress. We examined markers of oxidative stress (8-isoprostane and protein carbonyls), NAD(P)H-oxidase, xanthine oxidase and antioxidant enzyme (superoxide dismutase, catalase and glutathione peroxidase) activity in skeletal muscle of MCD patients (N = 12) and controls (N = 12). Eight-isoprostanes and protein carbonyls were higher in MCD patients as compared to controls (p < 0.05). There was a compensatory up-regulation of catalase protein content and activity (p < 0.05), mitochondrial superoxide dismutase (MnSOD) protein content (p < 0.01) and activity (p < 0.05) in MCD patients, yet this increase was not sufficient to protect the muscle against elevated oxidative damage. These results suggest that oxidative stress in McArdle patients occurs and future studies should evaluate a potential role for oxidative stress contributing to acute pathology (rhabdomyolysis) and possibly later onset fixed myopathy.

## 1 Introduction

McArdle disease (MCD) or Type V glycogen storage disease (OMIM #232600) is an autosomal recessive condition characterized by the absence of glycogen phosphorylase activity in skeletal muscle [1]. This enzyme is required for efficient glycogen breakdown during cellular energy need such as physical activity. Patients with this disorder often have exercise intolerance characterized by muscle pain and cramping during moderate- to high-intensity exercise as well as weakness and fatigability [1], [2], [3], [4], [5]. Additionally, more strenuous activities may lead to painful muscular contractures, rhabdomyolysis, and myoglobinuria [3], [5]. The basal level of serum creatine kinase (CK) activity, an indicator of skeletal muscle damage due to loss of cell membrane integrity [6], is chronically elevated in MCD patients as compared to their age- and gender-matched sedentary controls [7]. Older patients with MCD often develop a slowly progressive proximal myopathy and fixed weakness, however active patients have a better clinical outcome and functional capacity [8].

A lack of physical activity in MCD patients attenuates mitochondrial biogenesis and enzyme activity [9], [10]. Recently, it has been shown that moderate aerobic exercise training is well tolerated by MCD patients and leads to adaptations that increase oxidative capacity and health status [7], [11], [12], [13], [14]. It is well documented that physical activity induces physiological adaptations in healthy people including increased mitochondrial volume and content, and increased mitochondrial enzyme activities [15], [16], [17]. These adaptations may decrease oxidative stress in the following ways: (1) increased antioxidant enzyme content and/or activity, (2) reduced basal production of oxidants and (3) attenuation of reactive oxygen species (ROS) leakage during oxidative phosphorylation resulting in reduced oxidative damage to macromolecules [18], [19], [20], [21].

There are several possible reasons why MCD patients would have higher levels of oxidative stress. Forearm exercise testing is associated with an exaggerated elevation of ammonia, hypoxanthine and uric acid in MCD patients versus controls [22], [23], [24]. This observation is consequent to increased flux through the uric acid pathway beginning with myoadenylate deaminase and ending with uric acid formation by xanthine oxidase (XO), which contributes to myogenic hyperuricemia. An increased flux through XO would lead to higher superoxide anion and/or H_2_O_2_ generation and result in a higher level of oxidative stress. An increase in inflammatory cells in muscle following an acute bout of rhabdomyolysis could also lead to a transient increase in oxidative stress from invading neutrophils [25], [26]. Furthermore, rhabdomyolysis may perpetuate ROS generation by releasing myoglobin, which may act locally (muscle) and distally (kidney) to further induce macromolecular damage [27]. Finally, the repeated effects of varying degrees of rhabdomyolysis could contribute to the cumulative effect of normal aging associated oxidative stress [19], and produce a synergistic effect resulting in the later onset fixed myopathy seen in MCD patients. Recently, elevated levels of oxidative stress have been found in skeletal muscle of MCD patients [28]. We assume that oxidative stress can be a main cause of rhabdomyolysis in these patients (disruption of muscle fiber membrane and leakage of CK to the extracellular space). Therefore, higher oxidative damage in skeletal muscle of MCD patients may be one of explanatory mechanism because owing to their muscle metabolic deficiency.

The purpose of the present study was to characterize oxidative stress and a compensatory antioxidant enzyme responses in skeletal muscle of patients with MCD (glycogen phosphorylase deficiency) as compared to sedentary control patients. Based partially on the theory put forth by Russo and colleagues [29], and recent data [28], we hypothesized that higher oxidative stress occurs in skeletal muscle of sedentary MCD patients in association with: (1) higher levels of 8-isoprostanes and protein carbonyls, and (2) elevated antioxidant defenses as a compensatory response to chronic oxidative stress.

## 2 Materials and methods

### 2.1 Subjects

#### 2.1.1 MCD patients

Twelve subjects (N = 4 women and N = 8 men) with MCD (all reported exercise induced myalgia) were included in this study. Myophosphorylase activity was absent (histochemistry) or < 1% of normal activity (biochemistry) in all MCD patients and electron microscopic examination of muscle tissue specimens revealed elevated glycogen accumulation [30]. In addition, all patients had known mutations in the *PYGM* gene in *trans* or a known mutation and a predicted pathogenic mutation or two alleles containing novel sequence variants predicted to be pathogenic [31]. None of the subjects had experienced a bout of clinically relevant rhabdomyolysis in the 12 months before the muscle biopsy and none were participating in a physical activity program at the time of the biopsy.

#### 2.1.2 Control subjects

MCD patients (38.8 ± 10.8 y) were age- and sex- matched with control subjects (39.1 ± 10.8 y; N = 4 women and N = 8 men) who did not have MCD but were referred to the Neuromuscular and Neurometabolic Clinic for other reasons and were not clinically symptomatic for any other neuromuscular or neurometabolic disease. The control subjects had normal phosphorylase activity in muscle, normal electromyography and normal histology and ultrastructural assessment. A Likert-type scale was used to assess habitual exercise training status for each subject [32]. None of the patients were taking allopurinol. There was one smoker in each group (control and MCD), none had diabetes, and two MCD and two controls took 400 IU of vitamin E a day. None were taking anti-oxidant supplements (other than the subjects on vitamin E). All participants were working and independent in daily activity with no gait assistive devices. None of the subjects had fixed proximal weakness or myopathic/dystrophic changes in the skeletal muscle biopsy by histology (both controls and MCD). The training status of the two groups was similar indicating a sedentary activity level for both groups and no difference between them using a Student's 2-tailed unpaired *t*-test.

#### 2.1.3 Study design

This study was completed using extra muscle following all diagnostic testing on each participant and was approved after muscle collection by the McMaster University Hamilton Health Sciences Human Research Ethics Board and conformed to the Declaration of Helsinki guidelines. Written informed consent was obtained from all study subjects for the muscle biopsy. The muscle biopsy was taken from *vastus lateralis* muscle under local anesthesia in the morning after an overnight fast as described [33]. The tissue was immediately frozen in liquid nitrogen and stored at − 80 °C until analysis.

#### 2.1.4 RNA isolation

Frozen skeletal muscle tissues samples (25–40 mg) from all 24 participants were subjected to the Trizol method of total RNA extraction as per manufacturer's instructions (Invitrogen, Burlington, ON, Canada) and described by Mahoney et al. [34]. Selected samples were run on a denaturing agarose gel to verify RNA integrity. The ratio of the 28S to 18S rRNA was consistently > 1 for each sample selected indicating good quality RNA.

#### 2.1.5 Real time RT-PCR

Real time RT-PCR was performed using TaqMan chemistry (TaqMan One Step RT-PCR Master Mix Reagents, Applied Biosystems, Streetsville, ON, Canada) according to the manufacturer's instructions and previously described [34]. Beta (2) microglobulin (β2M) was used as a housekeeping gene to which target gene threshold cycle values were normalized. The use of β2M as a housekeeping gene for this study was validated by ensuring that its mRNA expression was not significantly different between MCD and controls (p = 0.2; data not shown).

#### 2.1.6 Muscle sample preparation

Frozen skeletal muscle tissue samples from subjects were homogenized in a 2 mL Tenbroeck homogenizer at 1:25 (wt/vol) in phosphate buffer (50 mM potassium phosphate, 5 mM EDTA, 0.5 mM DTT and 1.15% KCl at pH 7.4). Protease inhibitors (Sigma Chemical Co., St. Louis, MO) were added to the phosphate buffer immediately prior to use at a ratio of 1:1000 and 1 mM butylated hydroxytoluene was added to the samples that were designated for measuring 8-isoprostanes. Samples were centrifuged at 600*g* for 10 min at 4 °C and the supernatant was divided into serial aliquots for enzyme activity, western blot and markers of oxidative stress. Samples were frozen in liquid nitrogen and stored at − 80 °C. Protein content was determined using the method of Lowry and colleagues [35].

#### 2.1.7 Immunoblot preparation and analysis

Western blots were performed as previously described [36]. Briefly, 5–20 μg of protein were loaded per sample and heat denatured for 10 min at 90–99 °C. Following electrophoresis, the proteins were transferred onto nitrocellulose membranes and electroblotted for 1 h at 100 V. Membranes were blocked overnight with 5% dry milk in TBS with 0.1% Tween-20. Membranes were probed with the primary antibody and anti-actin (BD Biosciences, Mississauga, ON, Canada) antibody, as a loading control, for 2 h. The antibodies were purchased from Abcam (Cambridge, MA) and Santa Cruz (Santa Cruz, CA) and used at the following dilutions: catalase (AB16731) 1:1000; SOD1 (AB16831) 1:5000; SOD2 (AB13534) 1:3000; GPx1 (AB16798) 1:1000; p67^phox^ (SC15342) 1:100. A horseradish peroxidase-conjugated secondary antibody (Amersham, Piscataway, NJ) was used and visualized using enhanced chemiluminescence detection according to the manufacturer's instructions (ECL Plus, Amersham). Densitometry was performed on scanned images of X-ray film (Kodak XAR) using *ImageJ* v1.34s software.

### 2.2 Enzyme activity

#### 2.2.1 Superoxide dismutase (SOD)

Total SOD activity was determined in muscle by measuring the kinetic consumption of O_2_^−^ by superoxide dismutase in a competitive reaction with cytochrome

c as described by Flohe and Otting [37]. Briefly, 20 μL of supernatant were added to a cuvette containing 965 μL of medium (50 mM phosphate buffer, 0.1 mM EDTA, pH 7.8) with partially acetylated cytochrome *c* (25 mg/100 mL) and 0.5 μM xanthine. Fifteen microliters of XO (0.2 U/mL) were added to initiate the reaction, and absorption at 550 nm was measured for 3 min. One unit of SOD activity was defined as the amount of enzyme required to cause a 50% inhibition in cytochrome *c* reduction. In a separate cuvette, MnSOD activity was measured on the same sample and analyzed under identical conditions with the addition of 20 μL of 0.2 M KCN (prepared fresh at pH 8.5–9.5). The reactions were carried out at 30 °C in a temperature controlled Cary 300 Bio UV–Visible spectrophotometer (Varian, Palo Alto, CA). Cu/ZnSOD activity was calculated by subtracting MnSOD activity from total SOD activity. All of the samples were analyzed in duplicate. The SOD activities were expressed in U/mg protein.

#### 2.2.2 Catalase (CAT)

Muscle CAT activity was determined by measuring the kinetic decomposition of H_2_O_2_ according to Aebi [38]. Briefly, 40 μL of supernatant were added to a cuvette containing 950 μL of 50 mM phosphate buffer with 5 mM EDTA, and 0.05% Triton X-100 at pH 7.4. Ten microliters of 1 M H_2_O_2_ were added to the cuvette and mixed to initiate the reaction. Absorbance was measured at 240 nm for 2 min at 25 °C in a temperature controlled Cary 300 Bio UV–Visible spectrophotometer. All of the samples were analyzed in duplicate. CAT activity was expressed in μmol/min/mg protein.

#### 2.2.3 Glutathione peroxidase (GPx)

Muscle GPx activity was measured using Trevigen HT Glutathione Peroxidase Assay Kit (Trevigen, Cedarlane Laboratories Limited, Ontario, Canada) according to the manufacturer's instructions. All samples were run in duplicate and results were expressed in nmol/min/mg protein.

#### 2.2.4 Xanthine oxidase (XO)

Muscle XO was assayed using Amplex Red Assay Kit (Molecular Probes, Invitrogen detection technologies, Toronto, Canada) according to the manufacturer's instructions. All samples were run in triplicate and normalized to total protein content. Results were expressed in mU/mg protein.

#### 2.2.5 NAD(P)H oxidase

Activity of NAD(P)H oxidase was assayed by the reduction of NADH or NADPH as previously described [39]. Briefly, 25 μL (~ 100 μg of protein) of supernatant were added to the cuvette containing 955 to 965 μL of 50 mM phosphate buffer with 0.1 mM EDTA at pH 7.5, 100 μM NADH or 100 μM NADPH in the absence or presence of inhibitors of ROS-generation: 2 μM diphenyleneiodonium (DPI), 200 μM apocynin (APO) or 25 μM rotenone. The reduction of NAD(P)H was monitored by measuring the decline in absorbance at 340 nm over 30 min at 37 °C in a temperature controlled Cary 300 Bio UV–Visible spectrophotometer. All samples were analyzed in duplicate. NAD(P)H oxidase activity was expressed in nmol/min/mg protein.

#### 2.2.6 Mitochondrial isocitrate NADP dehydrogenase (mICDH)

Muscle mICDH activity was measured according to the method of Kil and colleagues [40] with slight modification. Briefly, 10 μL of supernatant were added to the cuvette containing 970 to 980 μL of 50 mM phosphate buffer, 0.1 mM EDTA, 5 mM MgCl_2_ and 0.05% Triton X-100 at pH 7.6 and 500 μM NADP. The reaction was started by the addition of 5 mM dl-isocitrate. The production of NADPH was monitored by measuring the increase in absorbance at 340 nm for 3 min at 30 °C in a temperature controlled Cary 300 Bio UV–Visible spectrophotometer. All samples were analyzed in duplicate. mICDH activity was expressed in nmol/min/mg protein.

### 2.3 Markers of oxidative stress

#### 2.3.1 Protein oxidation

Protein carbonyls were measured using an enzyme immunoassay (Zentech PC Test, Biocell Corp., Dunedin, NZ) with slight modification. All samples, standards, and quality controls were normalized to the same amount of protein content (protein concentration of the lowest sample), and 5 μL of each sample, standard, and control were then incubated in a final volume of 20.5 μL with dinitrophenylhydrazine (DNPH) for 45 min at room temperature. Following derivatization with DNPH, 5 μL of each sample (also standards and controls) were added into 1 mL of enzyme immunoassay buffer and the manufacturer's instructions were followed, starting with ELISA procedure. All samples were run in triplicate and results were expressed in nmol/mg protein.

#### 2.3.2 Lipid peroxidation

Muscle 8-isoprostane levels were measured using an EIA Kit (Cayman Chemical Company, Ann Arbor, Michigan, USA) according to the manufacturer's instructions. All samples were run in triplicate and normalized to total protein content and results were expressed in pg/mg protein.

The intra-assay coefficients of variation (CV) for markers of oxidative stress and enzyme activities were < 10%.

#### 2.3.3 Statistical analysis

We used one-tailed nonparametric Mann-Whitney *U* tests for 8-isoprostanes, protein carbonyls, and XO activity since a priori hypotheses were made such that these markers would be elevated in MCD patients as compared to controls. For all other analyses, we used two-tailed Mann-Whitney *U* test with the exception of non-mitochondrial NAD(P)H oxidase where we used Wilcoxon tests. A p-value of 0.05 or less was considered significant. Statistical analysis was performed using a software package (Statistica, V. 8.0, Tulsa, OK) and results are presented as mean ± SD.

## 3 Results

### 3.1 mRNA expression

The mRNA expression of CAT was lower in MCD patients as compared to control subjects (p < 0.05; Table 1). The mRNA expression of the genes for Cu/ZnSOD, MnSOD, GPx1 and XO were not different between MCD patients and controls (Table 1).

**Table 1:** The expression of mRNA levels in skeletal muscle of MCD patients and controls.

	mRNA expression (2-ΔCt ± SD)		mRNA expression (% changes ± SD)
	MCD	Control	MCD vs. control
CAT	0.11 ± 0.03	0.13 ± 0.03	80.9 ± 22.9*
Cu/ZnSOD	2.37 ± 0.87	3.34 ± 1.48	71.1 ± 26.1
MnSOD	0.81 ± 0.90	0.89 ± 0.68	91.4 ± 101.4
GPx1	0.02 ± 0.02	0.02 ± 0.01	110.9 ± 88.8
XO	7.259E-05 ± 9,002E-05	4,853E-05 ± 2,218E-05	149.6 ± 185.5

CAT mRNA was significantly lower in MCD patients than in controls (*p < 0.05).

### 3.2 Markers of oxidative damage

**Fig. 1:**
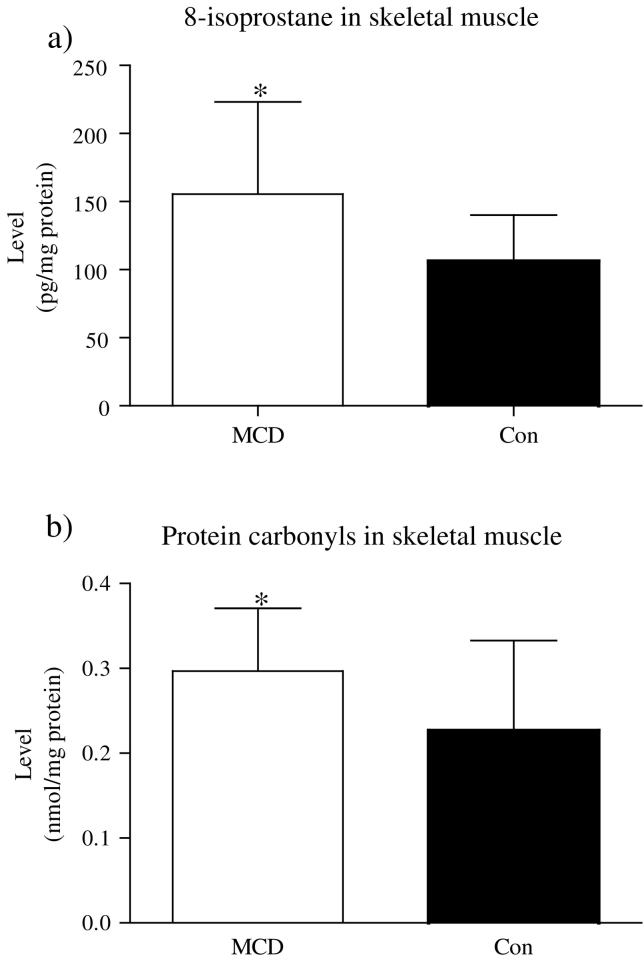
The level of markers of lipid and protein peroxidation in *vastus lateralis* muscle of MCD patients and controls. (A) 8-isoprostane level in skeletal muscle was higher in MCD (n = 10) as compared to Con (n = 12) (*p < 0.05). Results were expressed as pg/mg of protein. (B) The content of protein carbonyls was significantly elevated in MCD (n = 10) than in controls (n = 12) (*p < 0.05). Protein carbonyls were expressed as nmol/mg of protein.

Muscle eight-isoprostane content was 46% higher in MCD patients compared with controls (p < 0.05; Fig. 1A). Muscle protein carbonyl content was 31% higher in MCD patients (p < 0.05; Fig. 1B).

### 3.3 Enzyme activity and protein content

**Fig. 2:**
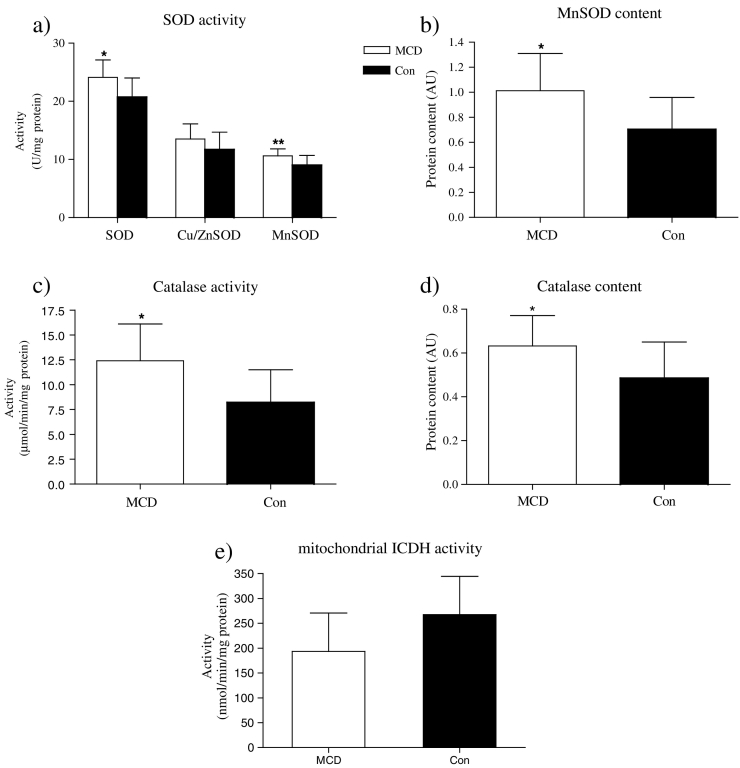
The enzyme activities in skeletal muscle of MCD and controls. (A) SOD activity in skeletal muscle was higher in MCD (n = 8) than in Con (n = 10) (*p < 0.02). Cu/ZnSOD activity was not different between MCD and Con. MnSOD activity was higher in MCD as compared to Con (**p < 0.05). Enzyme activities were expressed as U/mg of protein. (B) Catalase activity in skeletal muscle was higher in MCD (n = 8) than in Con (n = 11) (*p < 0.05). Enzyme activity was expressed as μmol/min/mg of protein. (C) The activity of GPx was not different between groups (n = 10). (D) Xanthine oxidase activity in MCD patients vs. Con (n = 9). (E) The activity of mitochondrial ICDH was not different in muscle of MCD (n = 10) and Con (n = 9). Enzyme activity was expressed as nmol/min/mg protein (C), (E) and as mU/mg protein (D).

Total SOD and MnSOD activities were higher in MCD patients (p < 0.02 and 0.05, respectively; Fig. 2A) with a trend toward higher Cu/ZnSOD activity (p = 0.12; Fig. 2A). There was higher CAT activity (p < 0.05; Fig. 2B) and elevated CAT protein content in MCD patients (p < 0.05; Table 2). There was no difference in GPx activity between MCD patients and controls (Fig. 2C). The activity of XO tended to be higher (p = 0.074; Fig. 2D) and mICDH had a tendency to be lower (p = 0.061; Fig. 2E) in skeletal muscle of MCD patients but did not reach the level of significance. The protein content for MnSOD was higher in MCD patients (p < 0.01; Table 2). However, Cu/ZnSOD, GPx and p67^phox^ protein content were not different between the two groups (Table 2).

**Table 2:** The protein content in skeletal muscle of MCD patients and controls.

	Protein content (AU)		Protein content (% changes ± SD)
	MCD	Control	MCD vs. control
CAT	0.63 ± 0.14	0.48 ± 0.16	130.5 ± 28.5*
Cu/ZnSOD	1.18 ± 0.52	1.08 ± 0.35	108.8 ± 48.3
MnSOD	1.07 ± 0.22	0.70 ± 0.25	152.3 ± 32.0**
GPx1	1.44 ± 0.83	1.08 ± 0.70	133.4 ± 76.8
XO	N/A	N/A	N/A
p67phox	3.42 ± 2.68	2.53 ± 0.94	135.2 ± 105.8

CAT and MnSOD protein content was significantly higher in MCD patients than in controls (*p < 0.05; **p < 0.01). Note: subunit of NADH-oxidase; N/A not analyzed. Protein content was expressed as arbitrary units (AU).

Non-mitochondrial NAD(P)H oxidase activity was higher with NADH as a substrate than NADPH (p < 0.05) in control subjects and (p < 0.01) in MCD patients (Table 3). The consumption of NADH by NAD(P)H oxidase was lower in skeletal muscle of control group when the inhibitor APO was used (p < 0.05; Table 3). DPI inhibited ROS generation in both MCD patients and controls (p < 0.01). When rotenone was used as an inhibitor for complex I, the consumption of NADH by NAD(P)H oxidase was lower than without rotenone in MCD (p < 0.01; Table 3).

**Table 3:** Effect of various inhibitors on NAD(P)H oxidase activity in skeletal muscle of MCD patients and controls.

	NADH	NADH + APO	NADH + Rot	NADH + DPI	NADPH
Con	12.15 ± 4.59	7.96 ± 3.46^⁎^	5.44 ± 2.05^⁎⁎^	0.13 ± 0.05^⁎⁎^	0.99 ± 0.72^⁎^
MCD	9.96 ± 2.57	6.91 ± 3.05	5.16 ± 1.33^⁎⁎^	0.12 ± 0.03^⁎⁎^	1.46 ± 0.78^⁎⁎^

NAD(P)H oxidase activity is expressed as nmol/min/mg protein. APO and Rot versus no inhibitor, DPI versus no inhibitor and NADPH as compared to NADH (^⁎^p < 0.05, ^⁎⁎^p < 0.01).

## 4 Discussion

The main findings of the present study were that patients with McArdle disease show evidence of increased oxidative stress (8-isoprostanes and protein carbonyls) and a compensatory up-regulation of antioxidant enzymes (MnSOD and CAT) in skeletal muscle. Together, these findings are supportive of the hypothesis of higher oxidative stress in MCD patients put forth by Russo and colleagues over a two decades ago [29] and recently published data [28].

Eight-isoprostanes and protein carbonyls are established markers of lipid and protein peroxidation, respectively. The elevation of these markers in MCD supports the hypothesis that oxidative stress is one of the factors that can compromise the integrity of components of muscle membranes and could partially explain the chronic elevation of muscle enzyme activity (CK) in the plasma of patients with MCD. Higher ROS generation induces structural changes in lipids and proteins, which could lead to sarcolemmal peroxidation damage and increase the permeability of the cell to ions. This could result in elevated sarcoplasmic Ca^2 +^ levels causing sustained contracture, protease and phospholipase activation and increased catabolic enzymes that could initiate muscle fatigue and cramping in MCD patients. It is also likely that a disruption in muscle membrane integrity in MCD patients is multi-factorial and interrelated given that myoglobin release per se can exacerbate oxidative stress [27], and represent a vicious cycle.

Likely as a compensatory response to the elevated levels of oxidative stress, we found higher activity of some of the antioxidant enzymes in skeletal muscle from MCD patients. The higher CAT protein content and activity, in the absence of changes in mRNA, is likely a consequence of post-transcriptional or/and post-translational modification in skeletal muscle. Moreover, we [30] and other groups have reported that changes in mRNA abundance were not always associated with directionally similar changes in protein content or enzyme activity [41], [42]. Thus several potential mechanisms exist to explain this phenomenon. Firstly, oxidative stress induces higher production of H_2_O_2_ in mitochondria and/or cytoplasm and activates CAT, which has a much greater affinity for H_2_O_2_ at higher concentrations as compared to GPx [43]. Secondly, the higher level of NADPH, which comes from non-mitochondrial dehydrogenases, may induce activation of CAT. NADPH is essential for the enzymatic function of CAT-mediated H_2_O_2_ decomposition [44], [45]. We did not detect differences at the GPx1 mRNA level, protein content and enzyme activity in skeletal muscle between both groups. This result may suggest that the concentration of H_2_O_2_ was too high to induce changes in both species, which is in concordance with a much greater affinity of GPx at lower concentrations of H_2_O_2_ [43]. Thirdly, and elevated nuclear factor erythroid 2-related factor 2 (Nrf2) protein content (a master regulator of antioxidant transcription) was recently shown in skeletal muscle of MCD patients [28].

**Fig. 3:**
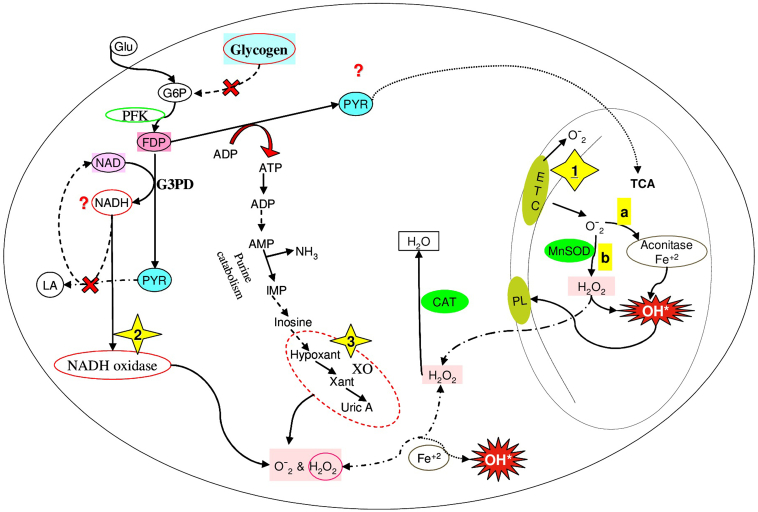
Proposed cellular sources involved in ROS generation in skeletal muscle in patients with an absence of glycogen phosphorylase activity. Elevated markers of lipid and protein oxidative damage and higher MnSOD, and CAT activity in skeletal muscle of MCD patients suggest several pathways involved in ROS generation: (1) Mitochondria: up-regulation of MnSOD implies higher production of superoxide anion (O_2_•^−^): a) O_2_•^−^ may react with Fe^+ 2^ from aconitase and produce hydroxyl radical (OH^⁎^). OH^⁎^ initiates free radical chain reactions in the phospholipids (PL) resulting in the release of reactive aldehydes, b) O_2_•^−^ may be dismutated by MnSOD or react spontaneously to H_2_O_2_. H_2_O_2_ can also produce OH^⁎^ and/or diffuse from the mitochondria to the cytosol where it is decomposed by CAT to water and oxygen, (2) Increased ROS generation could be mediated by non-mitochondrial NAD(P)H-oxidase by the up-regulation of phosphofructokinase (PFK). The higher production of NADH from glyceraldehyde-3-phosphate dehydrogenase (G3PD) could elevate NAD(P)H-oxidase activity in MCD patients as they do not “produce” lactate (LA). NADH must therefore be reoxidized and used in the G3PD reaction in glycolysis. Additional possibilities exist for NADH reoxidation: i) transport by malate-asparate shuttle and/or glycerophosphate shuttle, ii) activates CAT or iii) other mechanisms, (3) Significantly higher concentration of the purine metabolites in MCD patients than in controls following exercise suggested that XO would be elevated. We found only tendency of XO to be higher in mRNA level and activity in skeletal muscle of sedentary MCD patients. X indicates that MCD patients cannot breakdown muscle glycogen and cannot produce higher level of lactate during exercise.

There are important sources of ROS generation in skeletal muscle from MCD patients including the mitochondria [46] and cytoplasm [20], [47]. We assumed that one of the main sources of ROS generation in skeletal muscle of MCD patients would be XO, yet we found only a tendency for XO mRNA abundance and activity to be higher in MCD patients. The latter result is not too surprising given that the reaction is at equilibrium. Consequently, the lack of increase in activity is not an argument for a lack of importance given that the increased flux through this pathway (Fig. 3) is very well documented in MCD patients [22], [48]. It was found a much greater rise in ammonia following forearm exercise in MCD versus control patients in a previous study [49]. These results show that flux through the XO pathway is higher during exercise and chronically in MCD patients. Of potential therapeutic interest is the finding that allopurinol (an XO inhibitor) lowered oxidative stress markers in athletes during high intensity cycling [50]. However, in a recently, published study by Pareja-Galeano and colleagues did not support a central role of oxidative damage of skeletal muscle due to an eventual higher reliance on the XO pathways. The findings of above study were limited by the fact that markers of oxidative stress were not measured in skeletal muscle of MCD patients [51]. Thus, to further explore the role of the XO pathway and the genesis of oxidative stress and a potential MCD specific therapy, future studies should determine if oxidative stress is lowered with allopurinol during exercise in skeletal muscle of MCD patients.

The current study also found higher MnSOD protein content and activity in skeletal muscle in MCD patients. The inhibition of ETC complexes by rotenone and/or antimycin A results in enhanced ROS generation [52], [53] and MnSOD up-regulation [54]. Taken together, the lower activity of the ETC and deficient muscle mitochondrial respiration [9], [55], elevated Nrf2 protein content [28] and a strong trend for COX activity to be lower [30] in MCD patients, combined with elevated MnSOD protein content and activity in the current study, suggest that mitochondria are an important source of ROS generation in skeletal muscle of MCD patients (Fig. 3). Again, the changes in MnSOD activity and protein content in the absence of changes in steady state mRNA levels, imply that the compensation occurs at a post-transcriptional level. Mitochondrial glutathione (GSH) is critically important to mitigate ROS-mediated damage and is required for the activities of GPx and for mitochondrial phospholipid hydroperoxide glutathione peroxidase [56]. NADPH is an essential cofactor for the renewal of GSH, whereas glutathione reductase requires NADPH to maintain a favorable redox status of GSH. Furthermore, mICDH is a key enzyme in skeletal muscle defense against oxidative damage by facilitating regeneration of NADPH in the mitochondria [57]. There was a trend toward lower mICDH in skeletal muscle of MCD patients. This tendency implies higher ROS generation by mitochondria particularly in the context of a higher activity and protein content for MnSOD also seen in MCD patients.

In the current study we did not detect differences in activity of NAD(P)H oxidase between groups. The inhibitor, DPI, for ROS generation completely reduced NADH consumption by NAD(P)H oxidase in skeletal muscle of MCD patients and controls. APO (specific inhibitor for NAD(P)H oxidase) lead to decreased NADH consumption (67% inhibition) by the oxidase complex in both groups. We also did not find significant differences in p67^phox^ protein expression between groups. Overall, our results suggest that non-mitochondrial NAD(P)H oxidase was not a major cellular source of ROS generation in skeletal muscle of MCD patients. Although our finding showed that muscle non-mitochondrial NAD(P)H oxidase prefers NADH as a substrate (Fig. 3), which is in agreement with another report [39]. It is generally known that SOD is induced by superoxide anions; therefore, the higher SOD activity in skeletal muscle of MCD patients may reflect the generation of superoxide anions. It has also been documented that superoxide anions usually cannot cross biological membranes [58]. When these aspects are taken into consideration, the higher CAT protein content and activity may reflect an increased production of superoxide anions in the cytoplasm of MCD patients.

Sedentary MCD patients have elevated serum CK activity [7], [11]. It has been reported that aerobic training decreases serum CK activity in MCD patients [7]. In addition, it has been reported that aerobic training program significantly attenuated the impairment of skeletal muscle oxidative metabolism and improved variables associated with exercise tolerance [11], [59]. In contrast, deconditioning [60], immobilization [61] or age-related changes (decline of physical activity) [19], [62], lead to higher oxidative stress which may be very similar to MCD patients where the level of physical activity is low. It is interesting to speculate, and will be of interest to explore in the future, that part of the improvement in function and lower CK activity seen after training in MCD patients [7], is due to lower oxidative stress mediated by a compensatory up-regulation of MnSOD, and CAT activity as we have observed in older adults following an exercise training program [62].

In conclusion, markers of lipid and protein peroxidation were significantly higher in skeletal muscle of MCD patients as compared to the control group. The activity and protein content of MnSOD in MCD patients was higher than in controls. In addition, higher SOD and CAT activities were observed in MCD patients versus controls. We suggest that higher ROS generation in skeletal muscle can be a main cause of rhabdomyolysis in inactive MCD patients. The exact source of ROS in MCD patients is not fully elucidated but likely involves both mitochondrial and cytoplasmic generation pathways. Monitoring markers of oxidative stress will be useful in future studies where antioxidants and exercise are considered as a method of treatment for such patients.

## List of abbreviations

APO: apocynin
CAT: catalase
GPx: glutathione peroxidase
DNPH: dinitrophenylhydrazine
DPI: diphenyleneiodonium
H_2_O_2_: hydrogen peroxide
MDA: malondialdehyde
ROS: reactive oxygen species
O_2_•^−^: superoxide anion
SOD: superoxide dismutase
Cu/ZnSOD: cytosol superoxide dismutase
MnSOD: mitochondrial superoxide dismutase
TCA: tricarboxylic acid cycle
